# Economic analysis of smallholder dairy cattle enterprises in Senegal

**DOI:** 10.1007/s11250-022-03201-y

**Published:** 2022-06-29

**Authors:** Evaristo Mukunda Malenje, Ayao Missohou, Stanly Fon Tebug, Emelie Zonabend König, Joseph Owino Jung’a, Rawlynce Cheruiyot Bett, Karen Marshall

**Affiliations:** 1 Department of Animal Production, University of Nairobi, P. O. Box, Kangemi, 29053- 00625 Kenya; 2 Inter-State School of Veterinary Sciences and Medicine (EISMV), BP 5077 Dakar, Senegal; 3 International Committee of the Red Cross (ICRC), Ministries Road, Amarat, Juba, South Sudan; 4 Department of Animal Breeding and Genetics, Swedish University of Agricultural Sciences, Box 7023, 750 07 Uppsala, Sweden; 5 International Livestock Research Institute, P.O Box 30709-00100, Nairobi, Kenya

**Keywords:** Senegal, Livestock, Dairy-cattle, Milk, Smallholder, Net returns

## Abstract

Smallholder dairy production in Senegal is important to both livelihoods and food and nutrition security. Here, we examine the economic performance of smallholder dairy cattle enterprises in Senegal, using data from longitudinal monitoring of 113 households. The mean (and standard deviation) of the net returns (*NR*) per cow per annum (*pcpa*) was 21.7 (202.9) USD, whilst the NR per household herd per annum (*phpa*) was 106.1 (1740.3) USD. Only about half (52.2%) of the dairy cattle enterprise had a positive *NR*. The most significant income components were milk sale followed by animal sale, whilst the most significant cost components were animal feed followed by animal purchase. When households were grouped by ranking on *NR*_*pcpa*_, an interesting trend was observed: whilst the mean *NR*_*pcpa*_ showed a fairly linear increase from the lowest to highest *NR* groups, income and cost did not. Income and costs were both higher for the lowest and highest *NR* groups, in comparison to the intermediate *NR* groups. The mean *NR*s of households grouped by the main breed type they kept were not significantly different from each other, due to large variances within the breed groups. However, the mean total income and costs were significantly higher for households mainly keeping improved dairy breeds (*Bos taurus* or Zebu × *Bos taurus* crosses) in comparison to those keeping indigenous Zebu or Zebu by Guzerat crosses. This study highlights the highly variable (and often low) profitability of smallholder dairy cattle enterprise in Senegal. Further actions to address this are strongly recommended.

**Supplementary Information:**

The online version contains supplementary material available at 10.1007/s11250-022-03201-y.

## Introduction

Senegal is a country located in West Africa, with a population of about 15 million people of which 52.8% are rural according to 2019 estimates from (FAOSTAT 2021). Milk is a product of substantial nutritional and economical importance (Seck et al. 2016; Bernard et al. 2019; Chengat Prakashbabu et al. 2020; Craighead et al. 2021). Currently though, dairy imports in Senegal are high, with a 2019 milk import value (inclusive of milk in dried, fresh and in other forms) of over 43 million US Dollars (FAOSTAT 2021). Much of this import is as powdered milk, increasingly including mixtures of skim milk and vegetable fat, which is of concern due to nutritional quality and environmental sustainability (Duteurtre et al. 2020). Whilst local milk production still does not meet the national demand, it has shown significant growth, for example increasing by about 66% between 2008 and 2018 (FAOSTAT 2021). Cattle, goats and sheep contribute to local milk production, with cattle as the main contributor, accounting for 88% of the production in 2018 (FAOSTAT 2021).

The Senegalese government has prioritized development of the dairy sector. For example, the National Program for Livestock Development (PNDE) released in 2013 highlighted modernizing the milk value chain through interventions focused on genetic improvement, feed security and capacity building of farmers. Other government-supported initiatives have included the establishment of a National Dairy Committee, actions around milk collection, and dairy cattle artificial insemination campaigns (Seck et al. 2016). The dairy cattle artificial insemination campaigns promoted the use of exotic dairy cattle though subsidized artificial insemination and were operational intermittently between 1994 and 2012 (Seck et al. 2016).

Household cattle enterprises practising low-input low-output systems, using indigenous cattle breeds with low milk production potential, are common in Senegal. Due to the government initiatives described above, as well as private investment, there are also some household cattle enterprises more focused on dairy production keeping indigenous by exotic crossbred dairy cattle or, less commonly, pure exotic dairy cattle. A recent study, Marshall et al. (2020), compared the profitability of different household dairy systems, as defined by breed type kept and level of management, using a bio-economic model. This was based on data obtained from monitoring 220 dairy cattle keeping households, with collectively about 3000 animals, over an almost 2-year period. The keeping of crossbred indigenous zebu × exotic *Bos taurus* animals under improved levels of management was found to be the most net-beneficial and cost-beneficial, with notably a 7.4-fold higher net benefit and a 1.4-fold more favourable cost–benefit ratio in comparison to the common, traditional system of keeping indigenous zebu animals under low management.

Here, we present an additional analysis on the same dataset as the Marshall et al. (2020) study. Rather than using a bio-economic model to look at economic performance, we determine the actual net returns made by individual household dairy cattle enterprises over the time-period they were monitored. In addition, comparisons are made between households grouped by ranking on net returns and the main breed type they kept. The study aims to gain further insights into the economic performance of household dairy cattle enterprises in Senegal, to strengthen recommendations towards a profitable and sustainable smallholder dairy cattle sector.

## Materials and methods

### Study area description

This project contributes to a larger project, called Senegal Dairy Genetics Project (https://senegaldairy.wordpress.com/ and Marshal et al. 2016; Marshall et al. 2017; Marshall et al. 2020). The Senegal Dairy Genetics project was conducted in two regions within Senegal, namely Thiès (Khombole and Tivaouane departments) and Diourbel (Mbacke and Touba departments). Both regions are found in the Groundnut Basin (*Bassin arachidier*) which is one of the 7 distinct agro-ecological zones in Senegal. Due to the relatively suitable climate in this zone, it is home to Senegal’s highest human and livestock populations (Beal et al. 2015). As a result, in the two sites, there has been a variable magnitude of dairy production intensification, including the introduction of exotic breeds (mainly through the government AI programs) and better feeding schemes (Seck et al. 2016). The diversity of cattle breed types in these regions was one of the basis for the choice of their inclusion in the larger Senegal Dairy Genetics project (see also Tebug et al. 2018 and Marshall et al. 2020). The regions have a Sudano-Sahelian climate and are part of the agro-pastoral production system characterized by a short wet season spanning from June to October and a long dry season from November to May. Mean annual rainfall is relatively low, averaging 400 mm. Average temperatures range between 19 and 40 °C depending on the season (Laminou et al. 2020).

### Households

Two hundred and twenty (220) dairy cattle keeping households were included in the overall Senegal Dairy Genetics project. Cattle enterprises were defined as dairy if they produced milk for human consumption (though many could also be considered dual-purposes, as they also sold animals for meat). These households were selected, amongst other, to ensure a diversity of cattle breed types (Marshall et al. 2020). For the analysis presented in this paper, households were excluded if they went transhumant over the survey period (as it was not possible to collect full data over this period (51 households), if they did not have full data for other reasons (26 households), or if the household did not have any lactating animals over the survey period (14 households). Finally, sixteen (16) households were excluded for having a NR component (such as a cost or benefits) 3.5 standard deviations above or below the mean, so that these outliers would not affect the interpretation of trends in subsequent analysis. The final number of households considered for inclusion in this analysis was thus 113 (though for the analysis comparing breeds, 6 additional households were excluded for either not having a predominant breed type or keeping breeds other the main breed types being compared).

### Data collection

Data collection comprised baseline surveys (conducted May to July 2013) and longitudinal surveys (July 2013 to April 2015). During the longitudinal survey, households were visited 13 times, at approximately equal time intervals, with information collected for the time-period back to the previous visit. The mean for the number of days between rounds for a particular household was 47 and ranged between 18 and 89 days. Both baseline and longitudinal surveys collected a range of information, including information on economics (costs and benefits) of the household dairy cattle enterprise as well as animal level data, such as milk yield, reproductive events and animal movements (including purchases and sales). Trained enumerators in the local language of Wolof conducted all surveys. See Marshall et al. (2020) for more details, including links to the surveys and data.

### Calculation of economic performance of the household dairy cattle enterprises

Net returns (NR) and gross margins (GM) for each household dairy cattle enterprise were calculated based on household-level data collected through the surveys. NR considered all income and benefits, whereas GM only considered income and cash costs (see more details below). Both were calculated as per household herd per annum (phpa) and per cow per annum (pcpa). Data was collected in the local Senegalese currency (CFA) but presented here in US Dollars, USD, with a conversion factor of 580 CFA per 1 USD used.

NR_phpa_ and GM_phpa_ were calculated as below.

$${{NR}_{phpa}=\left[{I}_{milk sale,phpa}+{B}_{milk consumed,phpa}+{B}_{milk given away,phpa}{+I}_{animal sale,phpa}{+ {B}_{animals gifted in,phpa} +B}_{animals given away,phpa}+{I}_{other incomes,phpa}\right]-\left[{{OC}_{milk given away,phpa}+{OC}_{animals given away,phpa}+C}_{animal purchase,phpa}+{C}_{feed,phpa}+{C}_{hired labour,phpa}+{OC}_{household labour,phpa}+{C}_{health,phpa}+{C}_{housing,phpa}+{C}_{reproduction,phpa}+{C}_{loan repayment,phpa}+{C}_{water,phpa}+{C}_{other expenses,phpa}\right]}$$

$${{GM}_{phpa}=\left[{I}_{milk sale,phpa}{+I}_{animal sale,phpa}+{I}_{other incomes,phpa}\right]-\left[{C}_{animal purchase,phpa}+{C}_{feed,phpa}+{C}_{hired labour,phpa}+{C}_{health,phpa}+{C}_{housing,phpa}+{C}_{reproduction,phpa}+{C}_{loan repayment,phpa}+{C}_{water,phpa}+{C}_{other expenses,phpa}\right]}$$

Where income components (I) comprise that from the sale of milk and milk products (I_milk sale_), animal sale (I_animal sale_) and other incomes (I_other incomes_). Benefit components (B) comprised milk consumed by the household members (B_milk consumed_), milk given away to others for consumption (B_milk given away_) and animals gifted to the household as dowry or inheritance (B_animals gifted in_) those given away as inheritance, dowry or for ceremonies (B_animals given away_). Cash costs components (C) comprise the costs associated with the animal purchase (C_animal purchase_), animal feed (C_feed_), hired labour (C_hired labour_), health-care of the animals (C_health_), animal housing (C_housing_), cow reproduction (C_reproduction_), repayment of loans associated with the household dairy cattle enterprise (C_loan repayment_), water for the animals (C_water_) and any other expenses (C_other expenses_). Other costs (OC) comprised milk given away (OC_milk given away_), animals given away (OC_animals given away_) and household-labour (OC_household-labour_)). Note that milk given away and animals given away were included both as benefits and as costs: benefits because they are products of the farm and costs because the household members do not utilize them.

NR_pcpa_ was calculated as NR_phpa_ divided by herd size in cow years (similarly for GM_pcpa_). One cow year was considered a cow being in the herd for a full year (for example, 1 cow year could be equal to two cows in the herd for 6 months each). A cow was defined as a female of 2 years of age or greater.

Calculation of the different components is described below. In all cases, these were initially calculated per household herd over the specific time-period that household was monitored (which ranged from 481 to 565 days). The resultant values were then converted to an annual time-period. See also the supplemental data for more on the components.

I_milk sale_, B_milk consumed_, B_milk given away_, OC_milk given away_ milk quantities were recorded for each lactating cow, for morning and evening milking, each data collection day (test-day), with the sum of these giving test-day milk yields (milk suckled by calves was excluded). Missing morning or evening milk records were predicted as per ICAR (2017) using the Liu et al. (2000) modified method. The survey collected information on how the test-day milk was used, including whether it remained ‘fresh’ or was processed (for example, into ghee or curd) and whether the fresh or processed milk was sold, consumed by the household, given away to others for consumption, or wasted. When sales occurred, the sale price was recorded. This data was used to determine the total value of fresh and processed milk sold for each test day (as the number of units of a product sold multiplied by the sale price for that unit, summed over the different milk products). The value of milk and processed milk for each day between the test-days was determined as the average of the preceding and following test-days, with all daily values summed to give I_milk sale_ over the monitoring period. B_milk consumed_, B_milk given away_ and OC_milk given away_ were determined by the same process, with the fresh or processed milk valued at the same price as that which the household would have sold it. In the case of missing data on the sale price of fresh milk or processed milk for a particular survey round, the mode of the relevant sale prices from other rounds was used.

I_animal sales_, B_animals given away_ and OC_animals given away_;I_animal sales_ were calculated as the summed value of all animals sold, whether alive or after slaughter. The value of animals sold was the sale price of animals as given by the farmers less any costs associated with the sale (such as transport or brokerage). The value of the animals sold but who had a missing sale price was determined as the modal sale price of the animals of the same sex, age and breed type. The value of B_animals given away_ and OC_animals given away_ was determined analogously.

I_other incomes_ income from other sources (I_other income_), which was typically from taking care of animals belonging to other households, was summed over survey rounds.

C_animal purchase_, B_animals gifted in_;C_animal purchase_ was calculated as the summed value of all animals purchased including transportation costs and other purchasing costs. The value of animals purchased but who had a missing purchase price was determined as the modal purchase price of the animals of the same sex, age and breed type. The value of B_animals gifted in_ was determined in an analogous method.

C_feed_ was determined by summing all feed purchases, including the cost of transporting the feed to the households. In cases where a specific feed purchase was reported, but without a purchase price, the purchase price was determined as the modal feed price for that feed type and quantity.

C_hired labour_, OC_household-labour_;C_hired labour_ was determined based on the salary of hired labourer (and did not include other benefits labourers may receive, such as meals or accommodation). If the labourer also provided labour to other household activities outside of the dairy cattle enterprise, the labour cost was prorated accordingly. Household labour (OC_household-labour_) was valued based on hired labour costs. Specifically, the modal labour cost per hired labourer was determined across all households, and this was used to determine the household labour cost prorated for the time the household members contributed to the household dairy cattle enterprise.

C_health_ was determined as the total cost of all animal health care, whether preventative or curative. It was inclusive of medical supplies and payment of the service providers. Where there was missing data for C_health_, the modal cost for the same type of care, for the same animal type and number, was used.

C_housing_ was determined by summing depreciation costs and maintenance costs of animal houses and other structures used by a household for cattle keeping. Depreciation costs were calculated over 15 years (Marshall et al. 2020). Structure construction cost (on which the depreciation was based) was taken as reported by the farmer in the baseline survey round. This cost was prorated cattle only used part of the space. Where structure construction cost was missing, the mode cost of constructing the same structure type was used. For households renting structures for dairy, the rental price was used.

C_reproduction_ was the sum of all costs associated with cow servicing by AI and that for hiring a bull for natural mating. AI services provided by the state had zero cost.

C_loan repayment_ was the sum of interest incurred on loans taken out to support the household dairy cattle enterprise. Loan repayments were calculated using loan interest rates and grace periods for each loan.

C_water_ was determined by summing water purchase costs and their associated transport costs. Where purchased water was also used for purposes other than the household cattle enterprise, the purchase and transport cost was pro-rated accordingly.

C_other expenses_ comprised the cost of other expenses incurred concerning the household dairy cattle enterprise (C_other expenses_) other than those listed above, for example co-operative fees, and the cost of buying ropes and milking buckets.

### Comparisons between groups of households

Comparisons of economic performance were made between households grouped by (a) ranking on NR_pcpa_ and (b) main breed type kept. For NR_pcpa_, households were grouped into five groups of approximately equal numbers, referred to as groups 1 to 5, with group 1 having the lowest NR and group 5 the highest. For main breed type kept, households were grouped by the predominant breed type they kept (see below for how animals were assigned a breed type, and also note that most households kept a mix of breed types but with a predominant type). Comparison of means between the groups was performed using a one-way analysis of variance (ANOVA) with a significance level (alpha) of 0.05. For cases of significance differences, the post-hoc test Tukey’s honestly significant difference (HSD) was used to further explore mean differences, with a family-wise error rate of 0.05.

### Assignment of breed type

Cattle involved in the study were assigned a breed type based on either genomic information (via admixture analysis) or (for those animals not genotyped) farmer recall as described in Marshall et al. (2020). The breed types assigned were indigenous Zebu (IZ); Indigenous Zebu and *Bo*s *taurus* cross (IZ × BT), Indigenous Zebu and Guzerat cross (IZ × GZ), and High *Bos taurus* (HBT). Breed group assignment was based on proportions of ancient zebu (AZ), recent zebu (RZ), ancient taurine (AT) and recent taurine (RT). The IZ breed group comprised animals that were 0.88–0.99 AZ according to genomic (admixture) analysis or 1.00 AZ from recall. The IZ × GZ breed group comprised animals that were 0.39–0.86 AZ and 0.13–0.61 RZ from genomic analysis, or 0.50 to 0.75 AZ and 0.25–0.50 RZ from recall. The IZ × BT breed group comprised animals that were 0.38–0.84 AZ and 0.13–0.61 RT by genomic analysis or 0.50–0.75 AZ and 0.25–0.50 RT by recall. Finally, the HBT breed-group comprised animals that were 0.63–0.98 RT and 0.00–0.36 AZ from genomic analysis or 0.75–1.00 RT and 0.00–0.25 AZ and 0.75–1.00 RT by recall. The most common AZ breeds were Zebu Gobra and Zebu Maure (known for being well adapted to the local environmental conditions); RZ was mainly Guzerat (a tropical breed developed from Indian Krankej cattle and Brasilian Crioulo cattle of European origin, Peixoto et al. (2010), and RT predominantly Holstein Friesian and Montbéliarde (both bred for high milk production).

### Drivers of net returns

Multi-variable regression analysis was used to determine what other factors (outside those included in the *NR* calculations) affected NR_pcpa_ and NR_phpa_. See Table 1 for the list of independent variables included in the full model. We also considered including gender of the household head, but there were too few female headed households (7 in total). The final (reduced) model was determined using stepwise regression with backward elimination based on Akaike’s information criterion (AIC), in R Core Team (2021) and the MASS package (Venables and Ripley 1999).

**Table 1:** Variables included in the full model for the regression analysis of net returns

Independent variable	Class	Description^a^
Site	Discrete	2: Thies (56), Diourbel (57)
Level of education of the household head	Discrete	4: informal (18), elementary koranic (61), primary (13), post-primary (21)
Number of household members (adults and children)	Continuous	19 (9.6)
Number of adult household members (> 18 years)	Continuous	7 (4.3)
Main ethnic group of household members	Discrete	2: Wolof (91), Fulani (22)
Primary livelihood source	Discrete	4: crop production (32), dairy cattle production (26), non-agricultural business (39), agricultural business (16)
The average household income per annum, where respondents selected from income ranges	Discrete	2:720–1440 USD (33), 1440–2880 USD (80)
Main means of selling milk	Discrete	2: individual customers (83), market (46)
Land size used for dairy as told by farmer, hectares	Continuous	1.6 (3.4)
Information provider on dairy cattle keeping	Discrete	3: other farmers (76), veterinarian (37)
Artificial insemination use in the last 5 years before the survey	Discrete	2: yes (94), no (36)
Importance of dairy cattle keeping to the household in comparison to ten years year earlier	Discrete	3: more important (79), same importance (16), less important (34)
Record keeping on dairy (written and mental)	Discrete	2: yes (80), no (33)
Herd size in cow years	Continuous	12.5 (9.9)
Main breed type kept	Discrete	5: IZ (41), IZ × GZ (22), IZ × BT(35), HBT (9), MX (6)

*USD*, US dollars

*IZ*, Indigenous Zebu; *IZ* × *BT*, Indigenous Zebu and *Bo*s *taurus* cross; *IZ* × *GZ*, Indigenous Zebu and Guzerat cross; *HBT*, High *Bos taurus*; *MX*, mixed

^a^For discrete variables, given is the number of levels, their names and, in brackets, numbers within each level. For continuous variables given is the mean and, in brackets, standard deviation

## Results and discussion

### Contextual information

#### Households

Various characteristics of the households included in the study are given in Table 1. The majority of households (93.8%) were male-headed, with the remainder (6.2%) female-headed. Dairy production was named as one of the top three livelihood sources by 85.8% households. Other main livelihood sources were agricultural business (named by 70.8% households), non-agricultural business (54.0%) and crop production (53.1%). Household income was reported to be between 1440 and 2880 USD per annum for the majority (70.8%) of households, and between 720 and 1440 USD for the remainder. Households are associated with two main ethnic groups, namely Wolof (80.5%) and Fulani (19.5%). Household size was on average 19 (with a standard deviation of 9.6) when all households members, including children, were counted. The most common education level of household heads was attendance at elementary Koranic school (54.0%), followed by post-primary education (18.6%), informal education (16%) and primary education (11.5%).

#### Dairy cattle enterprises

Some key features of the household dairy cattle enterprises are given in Table 1 and the supplementary information (Online Resource 1). The percentage of households keeping IZ, IZ × BT and IZ × GZ as their main breed type was 36.3%, 31.0% and 19.5%, respectively. Only 8.0% of households kept HBT. Milk was mainly sold to individual buyers (73.5% of households) whilst the remainder sold their milk at markets. Most households (79.6%) practised grazing and supplementary (purchased) feeding, whilst other households only used purchased feeds (15.0%) or only grazed their animals (5.3%). The main types of supplementary feeds were concentrates and groundnut cake. Animals were also fed on crop residues, including groundnut (as haulms), cassava (as stems and peelings) and corn (as stover). Grazing was done on communal land free of charge. For cattle reproductive strategies, most of the households (70.8%) used natural mating, 22.1% used a combination of natural mating and AI and 7.1% used AI alone. Seventy-one percent or households kept records (either written or by memory) on their cattle. The majority (67.3%) of the households relied on other farmers for information on dairy cattle keeping.

## Net returns and gross margins from cattle keeping, across all households

**Fig. 1:**
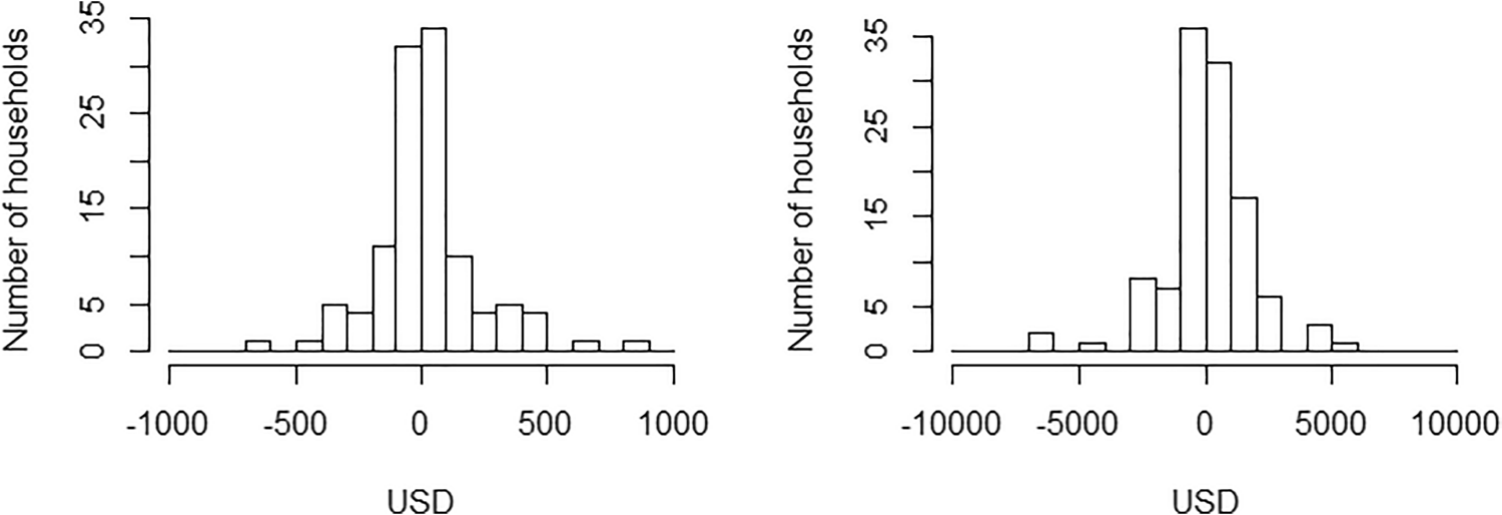
Distributions of net returns per cow per annum (left) and net returns per herd per annum (right), for all households

Results from the NR analyses are given in Fig. 1 and Table 2. For NR_pcpa_ and NR_phpa_, the mean and standard deviation (in brackets) was 21.7 (202.9) and 106.1 (1740.3) USD respectively. About half (52.2%) of the dairy cattle enterprises had a positive net return (even if small). The most significant income components (for both NR_pcpa_ and NR_phpa_) were milk sale followed by animal sale, whilst the most significant cost components were animal feed followed by animal purchase (Table 2). Gross margin (GM) analysis gave similar results to the NR analysis, as the value of benefits and non-cash costs was small (see Table 2). The correlations between NR and GM were high at 0.98 for pcpa and 0.99 for phpa: given this, the following results are presented for NR analysis only.

**Table 2:** Net returns and gross margin analysis, in US dollars

Variable	NR	GM	Per cow per annum				Per herd per annum			
			Mean	SD	Min	Max	Mean	SD	Min	Max
**Income and benefit components**										
Milk sale	∙	∙	172.2	178.6	1.4	910.7	1865.0	2794.5	12.5	20,962.9
Animal sale	∙	∙	120.3	192.5	0.0	1038.1	1002.8	1411.8	0.0	7896.1
Milk consumed	∙		19.5	18.7	0.0	85.7	182.2	173.3	0.0	817.5
Animals gifted in	∙		4.8	32.5	0.0	260.0	27.2	143.6	0.0	993.0
Animals given away	∙		3.1	14.9	0.0	131.9	27.5	124.7	0.0	1062.6
Milk given away	∙		2.5	6.6	0.0	39.2	30.0	85.5	0.0	713.1
Other incomes	∙	∙	0.4	4.6	0.0	48.5	3.1	31.1	0.0	329.9
**Total income NR**			**322.7**	**328.0**	**13.3**	**1562.6**	**3137.8**	**3935.4**	**160.9**	**30,817.2**
**Total income GM**			**292.9**	**302.5**	**13.3**	**1415.6**	**2870.8**	**3705.5**	**110.5**	**28,342.3**
**Cost components**										
Feed	∙	∙	146.4	194.3	0.0	1174.1	1393.1	2830.7	0.0	27,026.5
Animal purchase	∙	∙	62.8	119.7	0.0	679.6	831.1	1936.3	0.0	12,866.4
Hired labour	∙	∙	37.2	26.4	0.0	132.7	328.7	186.4	0.0	1064.4
Household labour	∙		20.9	21.1	0.0	93.3	167.8	153.9	0.0	833.9
Housing	∙	∙	11.3	19.9	0.0	129.0	102.0	154.6	0.0	798.8
Reproduction	∙	∙	6.6	15.8	0.0	104.4	45.0	97.4	0.0	574.3
Health	∙	∙	4.5	5.0	0.0	23.4	46.6	54.5	0.0	273.5
Water	∙	∙	4.3	5.6	0.0	27.8	39.4	51.4	0.0	218.5
Animals given away	∙		3.1	14.9	0.0	131.9	27.5	124.7	0.0	1062.6
Milk given away	∙		2.5	6.6	0.0	39.2	30.0	85.5	0.0	713.1
Loan repayment	∙	∙	1.1	6.6	0.0	54.7	14.8	99.4	0.0	921.1
Other expenses	∙	∙	0.5	2.0	0.0	18.0	5.7	33.1	0.0	330.0
**Total cost NR**			**301.1**	**287.7**	**16.1**	**1612.3**	**3031.8**	**4267.5**	**166.0**	**37,113.3**
**Total cost GM**			**274.6**	**278.7**	**4.4**	**1528.7**	**2806.4**	**4168.0**	**30.4**	**35,188.7**
**NR**			**21.7**	**202.9**	** − 639.1**	**807.4**	**106.1**	**1740.3**	** − 6590.1**	**5416.0**
**GM**			**18.3**	**195.3**	** − 602.6**	**806.0**	**64.4**	**1741.2**	** − 6846.4**	**5158.3**

*NR*, net returns; *GM*, gross margin; *SD*, standard deviation; *Min*, minimum; *Max*, maximum

**Fig. 2:**
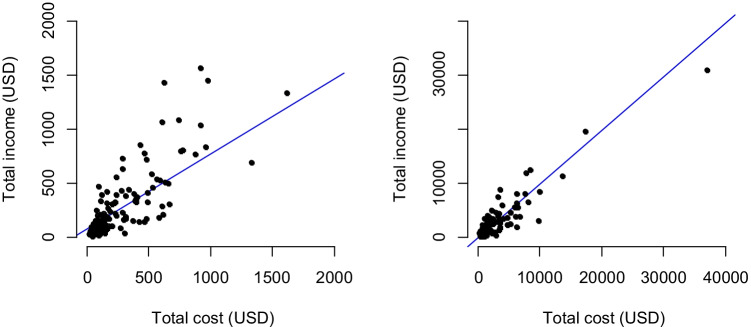
Total income versus total cost, for net returns (NR) analysis per cow per annum (left) and per herd per annum (right)

The correlation between total income and total cost for the household dairy cattle enterprises was high, at 0.79 and 0.91 in relation to the NR_pcpa_ and NR_phpa_ analysis, respectively, i.e., there was a tendency for households with the highest income to also have the highest costs (Fig. 2). It is also notable that many household clustered around low total income and low total cost. In addition, there are some outlier households (off the line of best fit) which are those with the highest NR (income greater than costs) and lowest NR (costs greater than income): these households are examined in more detail below.

A closer examination of outlier households showed that for the 3 households with the highest NR_pcpa_, NR seems to have been driven by high animal sale in 2 households and a combination of high animal and milk sale in the other household. For the 3 households with the lowest NR_pcpa_, animal sale was low in one household, animal purchase high in another households, whislt the 3rd household had a combination of both of these. There could be many interpretations for these events; for example, animal purchases could be as an investment to expand the dairy cattle enterprise, or alternatively, they could be to replace stock that had unintentionally exited the herd (such as through death). Similarly, high animal sales could be from emergency sales (due to the need of the household for cash), or planned as part of the business model. It is notable that the one limitation of this type of analysis is that it considers events within the time-frame of the monitoring period, which may not necessarily represent an ‘average’ time-period for that household.

### Households grouped according to NR_pcpa_

**Fig. 3:**
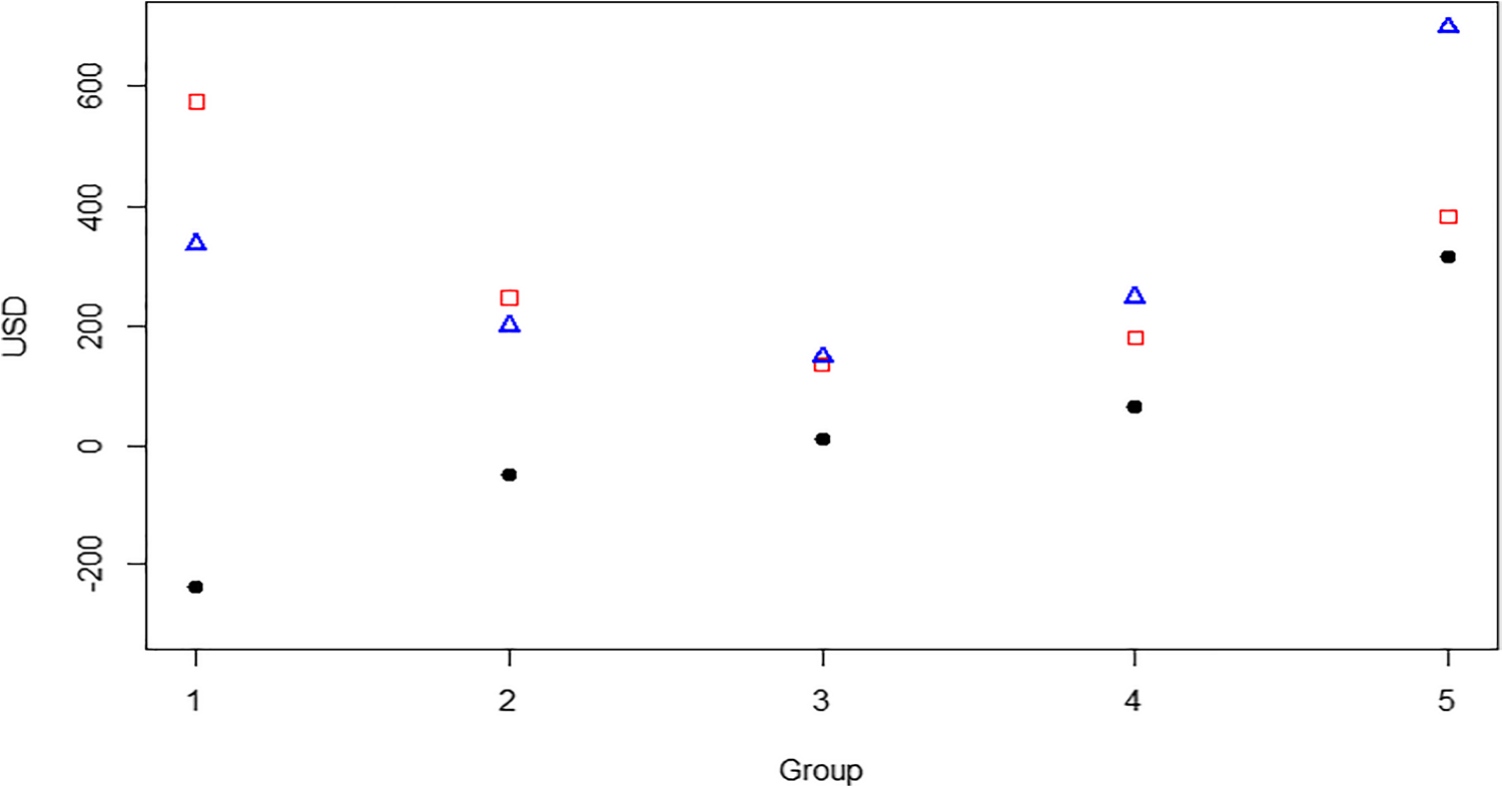
A graph showing total income per cow per annum (blue triangle), total cost per cow per annum (red square) and net returns per cow per annum (black circle) for groups of households formulated based on their ranking on net returns per cow per annum (with group 1 having the lowest net returns and group 5 the highest). USD, United States Dollars

To explore further the income and cost contributions to different levels of NR, households were grouped based on ranking on NR_pcpa_ (5 groups, group 1 with the lowest mean NR_pcpa_ and group 5 with the highest mean NR_pcpa_). See Table 3 and Fig. 3. It is interesting that whilst NR increased linearly from group 1 to group 5, the total income and total cost did not; rather, their plot showed a ‘U’ shape (Fig. 3). Group 1, with the lowest NR _pcpa_ (− 237.1 USD on average), had the highest costs (across the groups) and second highest income, whereas group 5, with the highest NR_pcpa_ (315.5 USD on average), had the highest income and second highest cost. The total income was statistically significantly higher for group 5 in comparison to all the other groups due to higher milk sale, milk consumption and/or animal sale (depending on the group its being compared to, see Table 3). The total cost was statistically significantly higher for groups 1 and 5 compared to the other groups, due to higher feed costs, animal purchase costs and/or labour costs (again depending on the group its being compared to). It is of note that the predominant breed type kept by group 5 households was IZ × BT, which was also the most net beneficial breed type identified in Marshall et al. (2020). Group 3 (with a NR _pcpa_ of 11.2 USD on average) had both the lowest total income and lowest total cost, i.e., these households practise a low-input low-output system. This fits with the dominant breed type kept by group 3 households being IZ (indigenous zebu) and the relatively large herd sizes, common features of cattle keepers in Senegal practising the traditional low-input low-output management systems. Also, of note is that whilst group 5 had a herd size lower than group 3 (9.0 compared to 17.7), it had higher income from animal sales (308.7 versus 36.6 USD): this may be due to group 5’s higher proportion of IZ × BT and HBT animals which have higher sale prices than the other breed-groups, particularly IZ (Marshall et al. 2020).

**Table 3:** Net return per cow per annum (NR_pcpa_) and herd structure, for household grouped based on NR_pcpa_ (group 1 has the lowest NR_pcpa_ and group 5 the highest). NR components are given in US dollars, as mean and standard deviation in brackets

	Group 1	Group 2	Group 3	Group 4	Group 5	*p*-value
*Net return analysis*						
**Income and benefit components**						
Milk sale	214.4(202.6)^a,b^	113.0(106.7)^b^	91.3(149.9)^b^	122.6(106.9)^b^	327.9(201.8)^a^	0.00⃰
Animal sale	93.4(153.1)^b^	71.3(102.1)^b^	36.6(33.4)^b^	98.5(98.6)^b^	308.7(321.3)^a^	0.00⃰
Milk consumed	19.9(20.2)^a,b^	13.9(17.3)^b^	12.7(14.5)^b^	21.2(18.5)^a,b^	30.6(18.5)^a^	0.09⃰
Animals given away	5.0(13.3)	0.0(0.0)	1.2(4.6)	2.5(11.8)	7.0(28.3)	0.52
Milk given away	4.3(10.3)	0.8(2.0)	3.0(8.3)	2.5(4.2)	1.9(4.6)	0.50
Animals gifted in	0.0(0.0)	1.0(4.7)	1.0(4.7)	0.2(1.0)	22.2(72.0)	0.09
Other incomes	0.0(0.0)	0.0(0.0)	2.1(10.1)	0.1(0.4)	0.0(0.0)	0.43
**Total income**	**336.9(321.2)**^**b**^	**200.0(177.4)**^**b**^	**147.9(164.3)**^**b**^	**247.6(174.7)**^**b**^	**698.2(416.3)**^**a**^	**0.00⃰**
**Cost components**						
Feed	306.8(246.7)^a^	99.8(116.2)^b^	56.0(128.2)^b^	66.7(110.6)^b^	212.3(215.5)^a^	0.00⃰
Animal purchase	143.0(164.9)^a^	59.0(92.8)^a,b^	21.7(51.8)^b^	35.5(64.3)^b^	58.0(153.8)^a,b^	0.06⃰
Hired labour	45.3(26.1)^a^	40.6(27.2)^a,b^	23.7(21.0)^b^	34.9(25.6)^a,b^	42.0(28.3) ^a,b^	0.05
Household labour	19.6(20.0)	23.6(24.0)	17.8(16.4)	20.4(19.4)	23.1(26.0)	0.88
Reproduction	17.0(26.7)^a^	5.0(10.0)^a^	0.8(2.5)^b^	0.8(2.3)^b^	10.0(17.2)^a^	0.00⃰
Housing	15.4(17.9)	9.8(16.5)	3.8(6.7)	10.8(21.8)	17.3(29.2)	0.18
Health	7.1(5.4)^a^	2.7(2.7)^b^	2.5(2.8)^b^	2.2(2.7)^b^	8.3(6.7)^a^	0.00⃰
Water	6.5(5.7)	5.7(6.9)	3.8(6.0)	2.9(3.9)	2.5(4.3)	0.07
Animals given away	5.0(13.3)	0.0(0.0)	1.2(4.6)	2.5(11.8)	7.0(28.3)	0.52
Milk given away	4.3(10.3)	0.8(2.0)	3.0(8.3)	2.5(4.2)	1.9(4.6)	0.50
Loan repayment	2.9(9.5)	0.1(0.6)	2.4(11.4)	0.2(0.8)	0.1(0.5)	0.41
Other expenses	1.2(2.3)	0.1(0.2)	0.1(0.2)	0.9(3.7)	0.1(0.3)	0.17
**Total cost**	**574.1(358.3)**^**a**^	**247.0(181.7)**^**b**^	**136.7(159.8)**^**b**^	**180.3(171.5)**^**b**^	**382.7(294.9)**^**a**^	**0.00⃰**
**Net returns**	** − 237.1(135.2)**^**d**^	** − 46.9(22.8)**^**c**^	**11.2(14.5)**^**b,c**^	**67.3(23.8)**^**b**^	**315.5(178.3)**^**a**^	**0.00⃰**
*Herd structure*						
**Herd size (cow years)**	**10.4(6.6)**^**a,b**^	**12.3(10.1)**^**a,b**^	**17.7(14.1)**^**a**^	**12.6(8.6)**^**a,b**^	**9.0(6.4)**^**b**^	**0.04⃰**
**Main breed type**	**Percentage of households per group**					
IZ	18.2	43.5	56.5	34.8	27.3	
IZ × GZ	22.7	13	26.1	21.7	13.6	
IZ × BT	31.8	39.1	13	30.4	40.9	
HBT	18.2	0	4.3	4.3	13.6	
MX	9.1	4.3	0	8.7	4.5	
*N* **of households**	**22**	**23**	**23**	**23**	**22**	

*N*, number; *IZ*, Indigenous Zebu; *IZ* × *BT*, Indigenous Zebu and *Bo*s *taurus* cross; *IZ* × *GZ*, Indigenous Zebu and Guzerat cross; *HBT*, High *Bos taurus*; *MX*, mixed

^*^Statistically significant at *p* ≤ 0.05. Means on the same row with different superscript letters are significantly different

Overall, these results suggest that there is a fine balance between households investing in the dairy cattle enterprises making a profit or not. It is also possible that households with a loss reported here will become profitable in later years, due to investments (e.g., in dairy cattle) paying off. In terms of targeting households for initiatives aimed at increasing local milk or meat production or profitability of smallholder cattle enterprises, both group 1 and group 5 households appear to be good candidates as these are investing in their household cattle enterprises.

### Households grouped according to breed type kept

In order to examine the effect of breed type on NR, the households were grouped based on the main breed type they kept, i.e., IZ, IZ × GZ, IZ × BT or HBT (Table 4). The number of households per group ranged from 41 for IZ to 9 for HBT: because of the low number of households in the HBT group, these results should be interpreted with care. The mean NR_pcpa_ for the different breed-groups was not statistically significantly different, due to the large variance around the means. However, there were statistically significant differences in total cost and income between the breed-groups. Total income was statistically significantly higher for HBT and IZ × BT, in comparison to IZ × GZ and IZ. This was due to differences in income from milk sale (as well as for milk consumed and given away) and animal sale. Of note is that income from milk sale was highest for HBT, followed by that for IZ × BT, and then IZ × GZ and IZ, which is as expected given that the *Bos taurus* breeds have been heavily improved for milk yield. The total cost was statistically significantly higher for HBT compared to IZ × BT, in-turn compared to IZ × GZ and IZ. This was due to differences in a number of cost components (Table 4), most notably feed costs and animal purchase costs. Feed costs were highest for HBT, followed by IZ × BT, and then IZ × GZ and IZ, suggesting that households with improved dairy breeds also invest more in feed. Additionally, it is of note that animal purchase costs were highest for HBT and BT × IZ, again as expected given the higher purchase cost of exotic or exotic-cross animals (Marshall et al. 2020).

**Table 4:** Net return analysis per cow per annum for household grouped based on main breed type kept, in US Dollars (mean and standard deviation in brackets). The number of households in each group is also given

	**IZ**	**IZ × GZ**	**IZ × BT**	**HBT**	***p*-value**
**Income and benefit components**					
Milk sale	81.0(83.1)^c^	103.0(138.9)^c^	245.9(149.7)^b^	453.5(283.1)^a^	0.00⃰
Animal sale	58.8(89.1)^b^	92.0(102.8)^a,b^	186.1(271.5)^a^	165.1(246.8)^a,b^	0.02⃰
Milk consumed	10.8(12.9)^c^	16.3(15.2)^b,c^	29.2(20.6)^a^	25.1(16.8)^a,b^	0.00⃰
Other incomes	1.2(7.6)	0.0(0.0)	0.1(0.3)	0.0(0.0)	0.68
Milk given away	0.8(2.1)^b^	3.9(5.7)^a,b^	2.1(6.7)^b^	9.0(15.4)^a^	0.01⃰
Animals gifted in	0.7(3.6)	0.0(0.0)	14.6(57.6)	0.0(0.0)	0.23
Animals given away	0.6(3.5)	3.9(10.4)	5.4(24.0)	5.1(15.4)	0.57
**Total income**	**153.8(141.4)**^**b**^	**219.1(185.8)**^**b**^	**483.3(363.8)**^**a**^	**657.9(511.6)**^**a**^	**0.00⃰**
**Cost components**					
Feed	59.6(95.0)^c^	83.7(94.5)^c^	212.9(181.5)^b^	399.9(389.3)^a^	0.00⃰
Hired labour	25.9(21.8)^b^	37.3(25.6)^a,b^	44.7(25.6)^a^	39.2(30.1)^a,b^	0.01⃰
Animal purchase	25.1(50.6)^c^	34.2(54.2)^b,c^	95.6(127.6)^a,b^	159.4(237.4)^a^	0.00⃰
Household labour	20.6(22.1)	29.5(24.4)	18.1(18.9)	13.4(16.2)	0.16
Housing	4.6(8.3)^b^	7.5(12.7)^a,b^	16.3(24.3)^a^	19.6(20.6)^a,b^	0.01⃰
Water	2.7(3.1)^b^	6.3(4.3)^a,b^	3.5(6.6)^a,b^	8.4(10.1)^a^	0.01⃰
Health	2.6(2.2)^b^	3.6(5.4)^a,b^	6.5(5.3)^a^	8.2(7.4)^a^	0.00⃰
Reproduction	0.9(3.7)^b^	4.0(7.8)^a,b^	12.4(22.6)^a^	12.5(22.0)^a,b^	0.06⃰
Other expenses	0.8(3.0)	0.1(0.3)	0.2(0.6)	0.3(0.4)	0.45
Milk given away	0.8(2.1)^b^	3.9(5.7)^a,b^	2.1(6.7)^b^	9.0(15.4)^a^	0.01⃰
Animals given away	0.6(3.5)	3.9(10.4)	5.4(24.0)	5.1(15.4)	0.57
Loan repayment	0.1(0.5)	0.0(0.1)	3.5(11.7)	0.3(0.8)	0.11
**Total cost**	**144.2(124.4)**^**c**^	**213.9(165.9)**^**c**^	**421.2(283.3)**^**b**^	**675.2(466.8)**^**a**^	**0.00⃰**
**Net returns**	**9.6(119.8)**	**5.2(128.3)**	**62.1(286.7)**	** − 17.4(272.4)**	**0.58**
***N* households**	**41**	**22**	**35**	**9**	

*N*, number; *IZ*, Indigenous Zebu; *IZ* × *BT*, Indigenous Zebu and *Bo*s *taurus* cross; *IZ* × *GZ*, Indigenous Zebu and Guzerat cross; *HBT*, high *Bos taurus*

^*^Statistically significant at *p* ≤ 0.05. Means on the same row with different superscript letters are significantly different

An economic comparison of the different breed types was also reported in Marshall et al. (2020), where a bio-economic model was utilised. Whilst this model was parameterised based on the same data as this study, various model assumptions were used (for simplification). For example, household herds were assumed to be of a constant size (not expanding or contracting) and of a single breed type (rather than a mix of breed types). Further animals (except breeding males) only entered the herd through birth, and cows were retained in the herd until culling age (i.e., no emergency sale). Despite these differences, the pattern of results found between the two studies aligns well. For example, Marshall et al. (2020) found total income, milk income, animal sales income, total costs and feed costs, to be highest for HBT, followed by IZ × BT, and then IZ × GZ and IZ, similar to that found here. The breed type with the highest net returns was found to be IZ × BT in the Marshall et al. (2020) study, which further aligns with that reported here (though in this study, this result was not statistically significant). A recommendation from this is to ensure that breed improvement programmes target production of cross-breed animals (IZ × BT): for this, the availability of cross-breed (IZ × BT) sires, or semen from them for input into artifical insemination, is key.

A key finding of the breed comparison reported here was the high variance in NR within a breed type. This indicates that not all households are equally benefiting from investment in improved breeds. Initiatives aimed at improving profitability of smallholder dairy or dual-purpose cattle enterprises, that are centred around the use of improved breeds, thus need to be careful to simultaneously address other issues that affect profitability.

### Drivers of net returns

Regression analysis was used to determine if there were any social or other factors, exogenous to those included in the economic analysis, which influenced NR_pcpa_ and NR_phpa_. Across all households, the best model to explain NR_pcpa_ had a coefficient of determination (*R*^2^) of 0.120, whilst that for NR_phpa_ had an *R*^2^ of 0.117. A relatively low *R*^2^ could be expected given the type of factors included in the model (i.e., exogenous to those including in the economic analysis, and only a limited sub-set of all possible factors included, as per data availability).

The final models were as follows:

$${NR}_{pcpa}=-39.0 + prior AI use (80.9 if yes, 0 if no) + milk buyer (101.5 if market, 0 if individual buyer) + site (-76.2 if Thi\grave{e} s, 0 if Diourbel)$$

$${NR}_{phpa}=-920+prior AI use \left(783.4 if yes, 0 if no\right)+milk buyer (1168.6 ifmarket, 0 if individual buyer)$$

For smallholder dairy cattle enterprises in Senegal, prior use of AI and selling milk at the market (versus to an individual buyer) increased the expected NR for both pcpa and phpa. That prior AI use and selling milk at the market had a positive effect on NR (whether pcpa or phpa) may relate to these household being more commercially orientated. Site was retained in the final model for NR_pcpa_ (though not NR_phpa_), with households in Thies having a lower NR_pcpa_ than households in Diourbel. The reason for this requires further investigation.

### Methodological approach and assumptions

This study determined the net returns for individual household dairy cattle enterprises over a set monitoring period, from detailed recording. The key advantages of this type of study are that it reflects ‘real-life’ (rather than a simplification as is often used in models) and that by grouping households the variation in responses can be observed which is important in relation to formulating recommendations on intervention options. For example, households who are risk averse may not wish to take up an intervention whose gains, although favourable on average, may be disadvantageous for some. Limitations of this approach are the heavy data requirements, inability to cleanly focus on individual interventions (for example adoption of a particular breed type, given farmers mostly had herds of mixed breed types) and inability to average over many years (excepting cases where monitoring has been very long-term, which is resource intensive). Several assumptions were made as part of this study, notably that missing values for a particular income or cost items were estimated as the mean or mode (as appropriate) from other relevant data. These assumptions may mean that the actual NR or GM achieved by households over the survey period varied from that calculated, but these differences are likely to be small.

## Conclusion

The Senegalese dairy sector faces many challenges, key amongst them being ease of importation of cheaper dairy products from Europe and the difficulty in increasing local milk production due to the harsh environment (Seck et al. 2016; Marshall et al. 2020; Craighead et al. 2021). Given the increasing need for the Senegalese dairy sector to be more efficient amidst these challenges, this paper adds insight into the economics of the smallholder dairy cattle enterprises. Of note is the high variance in net returns across the households, and that many households (close to 50%) did not make a profit from their dairy cattle enterprise over the monitoring period. Whilst it is recognized that smallholders keep livestock for multiple reasons and not just income (ILRI 2019), profitable livestock enterprises can be a key incentive for livestock keeping households to invest further in them. Further actions aimed at increasing the profitability of smallholder dairy cattle enterprises, and reducing risk in dairy-cattle keeping, are thus strongly recommended.

## Supplementary Information

Below is the link to the electronic supplementary material.

Supplementary file1 (PDF 300 KB)

## Data Availability

The datasets analysed during the current study are available in the ILRI repository, https://data.ilri.org/portal/dataset/sdgbaseline, https://data.ilri.org/portal/dataset/sdglong and https://data.ilri.org/portal/dataset/sdgmon.
