# Supplementary material for: Economic analysis of smallholder dairy cattle enterprises in Senegal
Source: Trop Anim Health Prod. 2022 Jun 29;54(4):221. doi: 10.1007/s11250-022-03201-y (PMC9242928; doi:10.1007/s11250-022-03201-y)
Supplement: Supplementary file 1 — Supplementary file1 (PDF 300 KB) [file 11250_2022_3201_MOESM1_ESM.pdf]

Supplementary information

Caption: Additional information on the components used to calculate net returns and gross margins.

Article title: Economic analysis of smallholder dairy cattle enterprises in Senegal

Journal name: Tropical Animal Health and Production

Author names: Evaristo Mukunda Malenje, Ayao Missohou, Stanly Tebug, Emelie Zonabend König, Joseph Owino Jung'a, Rawlynce Cheruiyot Bett, Karen Marshall

Affiliation and e-mail address of the corresponding author: Department of Animal Production, University of Nairobi, P. O. Box 29053- 00625, Kangemi, Kenya. malenje2012@gmail.com

$I_{milk\ sale}$, $B_{milk\ consumed}$, $B_{milk\ given\ away}$, $OC_{milk\ given\ away}$.

Over the survey period, all households (100%) had a milk sale value. The percentage of households which consumed and gave away some milk was 93.8% and 35.4% respectively. The mean and standard deviation (in brackets) of $I_{milk\ sale,\ pcpa}$ and $I_{milk\ sale,\ phpa}$ was 172.2 (178.6) and 1865 (2794.5) USD respectively. Analogous values for $B_{milk\ consumed}$ were 93.8%, 20.8 (18.6), 194.2 (172.3), and those for $B_{milk\ given\ away}$ and $OC_{milk\ given\ away}$ were 35.4%, 7.0 (9.6), 84.9 (127.4). Hand milking was used on all cows. Although calves were allowed to suckle the lactating cows for one to two minutes prior before milking (to stimulate let-down) and then after milking, this was not valued in the analysis performed here. Milk was sold as fresh milk or as processed milk at mode price of 0.8 USD (500CFA) and 1.0 USD (600CFA) per litre respectively.

$I_{animal\ sales}$, $B_{animals\ given\ away}$, $OC_{animals\ given\ away}$

76.1% of household sold one or more animals over the survey period. For these households, the mean and standard deviation (in brackets) of $I_{animal\ sale,\ pcpa}$ and $I_{animal\ sale,\ phpa}$ was 158.1 (206.9) and 1317.6 (1485.5) USD respectively. The number of animals sold per household ranged between 1 and 14 animals with the majority (75.6%) of the households selling less than 5 animals. Considering animal type, 34.3% of animals sold were cows (with a sale price range of 146.6 to 3448.3 USD), 20.9% young males (172.4-1120.7 USD), 17.2% bulls (129.3-2586.2 USD), 11.9% young females (172.4=2069 USD), 7.8% male calves (129.3-344.8 USD) and 7.8% female calves (163.8-577.6 USD). 7.1% of households gave one or more animal away over the survey period. For these households the range of $B_{animals\ given\ away}$ and $OC_{animals\ given\ away,}$ for pcpa and phpa, was 4.5 to 131.9 USD, and 176.8 to 1062.6 USD, respectively.

$I_{\text{other incomes}}$

Other income sources (to those listed above) were recorded by few households (2.75). For these households the range $I_{\text{other incomes, pcpa}}$ and $I_{\text{other incomes, phpa}}$ was 0.1 to 48.5, and 2.4 to 329.9 USD, respectively.

$C_{\text{animal purchase}}$, $B_{\text{animals gifted in}}$

Almost half (47.8%) of households purchased an animal over the survey period. The mean and standard deviation (in brackets) of $I_{animal\ purchase,\ pcpa}$ and $I_{animal\ purchase,\ phpa}$ was 131.4 (145.3) and 1739.1 (2512.7) USD respectively Of the households that purchased some animals, 75.9% purchased less than 5 animals, 20.4% purchase between 6 and 19 animals, and 3.7% purchased more than 10 animal. Considering animal type, 43.3 % of animal purchases were cows (with a purchase price ranging from 241.4 to 3103.4 USD), 17.5% young females (129.3 to 2241.4 USD), 17.5% male calves (86.2 to 431.0), 11.9% young males (129.3 to 1448.3 USD), 6.7% female calves (86.2 to 775.7 USD) and 3.1% bulls (327.6 to 862.1USD). Few (4.4%) of households received animals as gifts, dowry or inheritance, For these households the range $B_{\text{animals gifted in}}$was 4.6 to 260.0 USD for pcpa, and 154.5 to 993.0 USD for phpa.

C_{feed}

Almost all (95.6%) of households purchased feed over the survey period (most commonly to supplement grazing). For these households, the mean and standard deviation (in brackets) of $C_{feed,\ pcpa}$ *and* $C_{feed,\ phpa}$ was 153.1 (196.1) and 1457.6 (2879.6) USD respectively. Salmon et al. 2018 has documented further information on main feed types in this study and the cost of the different feed types.

$C_{hired\ labour}$, $OC_{household\text{-}labour}$

On labour provision, the majority (91.2%) of households used a combination of hired and household labour, whilst fewer households used only household labour (7.1%) or hired labour (1.8%). For households using hired labour the mean and standard deviation (in brackets) of $C_{\text{hired labour, pcpa}}$ and $C_{\text{hired labour, phpa}}$ was 40.0(25.2) and 353.7(168.8) USD respectively, whilst for households using household labour analogous values were 21.3 (21.1) and 170.8(253.6) USD respectively.

C_{health}

Most households (93.8%) recorded one or more curative or preventative health-care event. The mean and standard deviation (in brackets) of $C_{health, pcpa}$ and $C_{health,, phpa}$ was 4.8 (5.0) and 49.6 (54.9) USD respectively. Animal diseases caused by bacteria, viruses and parasites were recorded by 60.2%, 24.8% and 15.0% of the household respectively. Most households (77.9%) recorded curative and preventive animal healthcare events. The main animal-types treated were cows (39.1% of all treatment cases) followed by calves (30.2%).

$C_{housing}$

An animal housing cost was incurred by 88.5% of households with, for these households, a mean and standard deviation (in brackets) of $C_{housing, pcpa}$ and $C_{housing, phpa}$ of 12.8 (20.8) and 115.3 (159.7) USD respectively.

$C_{reproduction}$

About one-third (32.7%) of households incurred a cow reproduction cost. Natural mating and private AI was paid for by 10.6% and 7.1% of the households respectively while 2.7% of the households benefited from free AI by the state. For households incurring a reproduction cost, the mean and standard deviation (in brackets) of $C_{reproduction, pcpa}$ and $C_{reproduction, phpa}$ was 20.2 (22.3) and 137.4 (128.3) USD respectively. The modal cost of a single AI service was 69.0 USD, and ranged between 43.1 USD to 94.8 USD, whilst the modal cost of a single natural mating service was 43.1 USD and ranged between 34.5 USD to 43.1 USD.

$C_{loan\ repayment}$

Loans were repaid by few (7.1%) of households over the survey period. For these households the cost of repayment was 0.4 to 54.3 USD for $C_{loan\ repayment, pcpa}$ and 11.0 and 921.1 USD for $C_{loan\ repayment, phpa}$. The sizes of the loans ranged from 172.4 to 8620.7 USD, with 10 to 12 month repayment periods and interest rates of 5 to 16%,

C_{water}

Water costs were incurred by 63.7% of households, whist the remaining 36.3%of households obtaining water free of charge from tap, a creek, or a well either on or off their farms. For households paying for water, the mean and standard deviation (in brackets) of $C_{water, pcpa}$ and $C_{water, phpa}$ was 6.7 (5.8) USD and 61.9.4 (52.5) USD respectively. The water purchase cost for most households (89.8%) was less than 0.35 USD per 100 litres.

$C_{other\ expenses}$

Other expenses to those listed above were incurred by 21.2% of households. For these households the mean and standard deviation (in brackets) of $C_{\text{other expenses, pcpa}}$ and $C_{\text{other expenses, phpa}}$ was 2.2 (4.0) and 27.0 (68.8) USD respectively.
